# Exercise Intervention Associated with Cognitive Improvement in Alzheimer's Disease

**DOI:** 10.1155/2018/9234105

**Published:** 2018-03-11

**Authors:** Meng Ying Cui, Yang Lin, Ji Yao Sheng, Xuewen Zhang, Ran Ji Cui

**Affiliations:** Jilin Provincial Key Laboratory on Molecular and Chemical Genetic, The Second Hospital of Jilin University, 218 Ziqiang Street, Changchun 130041, China

## Abstract

Alzheimer's disease (AD) is a progressive neurodegenerative disease with the syndrome of cognitive and functional decline. Pharmacotherapy has always been in a dominant position for the treatment of AD. However, in most cases, drug therapy is accompanied with clinical delays when older adults have suffered from cognitive decline in episodic memory, working memory, and executive function. On the other hand, accumulating evidence suggests that exercise intervention may ameliorate the progression of cognitive impairment in aging ones while the standard strategy is lacking based on different levels of cognitive decline especially in mild cognitive impairment (MCI) and AD. MCI is the preclinical stage of AD in which neurodegeneration may be reversed via neuroplasticity. Therefore, taking exercise intervention in the early stage of MCI and healthy aging at the risk of AD could slow down the process of cognitive impairment and provide a promising cost-effective nonpharmacological therapy to dementia.

## 1. Introduction

Demographic changes make dementia an intractable social problem, and strategies for maintaining cognitive function with the growth of the age are seriously needed. Older adults complain of subjective gradual cognitive decline, and some of them are likely to develop dementia without efficient intervention. Alzheimer's disease (AD) and other dementias have a huge impact on the people with the disease, and taking care of them is a huge task for their families. Alzheimer's disease is becoming the fifth leading cause of death in people older than 65 years [1], and there will be 115 million people worldwide who will suffer from AD by 2050 [2]. Currently, AD is diagnosed through insoluble amyloid *β*-peptide (A*β*) in extracellular plaques and agminated tau protein in the intracellular neurofibrillary tangles by the detection of postmortem [3, 4].

Pharmacological and exercise therapies are the major intervention targets of AD. Cholinesterase inhibitors (donepezil, galantamine, and rivastigmine), memantine, and vitamin E have shown at least some efficacy in cognitive and functional decline [5, 6]. However, there has been no definite evidence in favor of the appropriate duration and the progression of disease when drug discontinuance. Drug treatment is always a challenge because of its great cost obviously. On the other hand, a growing base of evidence has indicated that exercise interventions enhance neuroplasticity, improve cognitive function and the ability of daily life, and reduce rates of neuropsychiatric symptoms. The mechanisms of exercise for improving cognition appear to be complex and unclear, including increased blood volume and capillarization [7, 8], decreased reactive oxygen species (ROS) and oxidative stress [9], reduced A*β* load and the levels of hyperphosphorylated tau proteins [10, 11], the modulation of cholinergic system [12], and the regulation of expression of brain-derived neurotrophic factor [13]. A six-month randomized trial using resistance training and aerobic training finds functional changes in three regions of cortex and hemodynamic activity in the lingual gyrus [7]; Guiney et al. confirmed that exercise-related cognitive benefits point to cerebral blood flow (CBF) and cerebrovascular regulation [8]; Smith et al. found a significant decrease in activation intensity of eleven brain regions by fMRI after a 12-week supervised treadmill walking, which suggests exercise intervention enhances neural efficiency [14]. In addition, exergaming leads to an increase of brain-derived neurotropic factor (BDNF) after cyber cycling [15], indicating that exercise benefits cognition by modulating neuronal plasticity through the regulation of dose-dependent BDNF [16]. Exercise-induced reduction of inflammatory markers (CRP, TNF-*α*, and IL-6) is probably one of its molecular mechanisms as well. These trials have generally demonstrated exercise a promising treatment for AD. This review (1) highlights the importance of exercise intervention on delaying the onset or slowing the progression of cognitive decline with different stages of Alzheimer's disease, (2) compares different types of exercise intervention on their role of cognitive function, (3) expounds the underlying mechanisms of exercise intervention on cognitive improvement, and (4) provides a structured strategy of exercise for clinical benefit.

## 2. Type of Exercise Intervention

The risk of developing AD increases exponentially with age, which affects daily life seriously and brings a heavy burden to each family. Therefore, there is an urgent demand to seek a therapy to at least ameliorate the progression of its cognitive disorder. Recently, exercise seems to be an emerging intervention as epidemiological and clinical studies suggest the positive effects of exercise training on cognition and counteracting the detrimental effects of neurocognitive illnesses. However, there is no consistent agreement of standard strategies for exercise intervention, and a better understanding of what type of exercise is most beneficial for cognitive performance is required especially.

### 2.1. Cognitive Exercise

There is some significant evidence to support claims of effectiveness of cognitive exercises including cognitive training (CT), cognitive stimulation (CS), and cognitive rehabilitation (CR) that differ in tasks. CT majors in standard tasks; extended cognitive practice training interventions have shown its role on enhancing performance in untrained cognitive tasks [17], and training resulted in improved targeted cognitive abilities (reasoning and speed) for 10 years [18]. A RCT illustrated that 10 sessions of occupational therapy improved functional capacity and helped AD patients adapt to their cognitive limitations through compensatory strategies [19]. However, the benefits of cognitive tasks cannot transfer flexibly and efficiently when accepters come to dissimilar tasks [20, 21]. CS refers to a wider range of activities that lack standardization, which aimed for general improvement in cognitive functions like puzzles, word games, and indoor gardening [22], while CR centers on a settled goal such as reasoning exercises, which resulted in less functional descent in self-reported activities of daily living [23, 24]. Nevertheless, opinions also divided into whether cognitive exercises induced only limited effects in executive function. Basak et al. found better performance in task switching, reasoning, working memory, and visual short-term memory after a real-time strategy video game, which indicates the enhancement of executive control process [25], while other team insists training older adults with nonaction video games enhances attention, immediate and delayed visual recognition memory, and processing speed except working memory (WM) and executive control [26].

### 2.2. Physical Training

More and more evidence from epidemiological, cross-sectional, and RCTs confirm that physical training such as aerobic [27–32] and resistance training [33–38] is associated with a reduced risk of cognitive decline. Aerobic exercises gain benefits by improving brain function mainly. In the early animal studies in 1995, Cotman and colleagues confirmed that a short-term wheel running for two days resulted in increased brain-derived neurotropic factor (BDNF) in the hippocampus and caudal cortex, which promotes the function of neurons [39]. Moreover, studies have demonstrated that aerobic exercises attenuate age-related myelin declines of corpus callosum [40] and remain white matter integrity [41] via better cardiorespiratory fitness. Higher levels of aerobic fitness alleviate hippocampal decay, increase hippocampal volume, and obtain better spatial memory performance as a consequence [29]. Those who participate in higher levels of physical exercises perform significantly better on global cognition and executive functioning [30–32]. On the other hand, resistance training works as an approach for cognitive enhancement as well. Twelve months of once or twice weekly resistance training benefited gait speed and decreased the risk for falls, which affects the daily life seriously [33]. However, different from aerobic-based exercise training, the mechanisms of resistance training focus on biomarkers which impact on neural functions such as insulin-like growth factor 1 (IGF-1) [34, 42] and serum homocysteine. Cassilhas et al. brought in sixty-two elderly individuals, let them participate in either moderate- or high-intensity resistance training, and found that the levels of IGF-1 which promotes neuronal growth were higher than those of the control group [34]. To the contrary, homocysteine decreases after resistance exercise training and obtains positive results because of its negative effects on neural function in the elderly [43]. Meanwhile, the relationship between Tai Chi (one of the typical modes of resistance training) and cognition was carried out in many studies. Chang and Wayne's group have gained positive results [35, 36] while Snowden et al. showed limited evidence of cognitive benefits [44]. Furthermore, a series of exercise programme comprised of resistance and balance training exercises was tested in many studies. Brown and coworkers verified that a programme composed of group-based exercise improved cognitive ability of fluid intelligence significantly [37]. Home-based strength and balance retraining was confirmed effective in executive functioning by Liu-Ambrose et al. [38]. However, different meta-analyses and systemic reviews have controversial opinions about physical intervention. A meta-analysis in 2014 found no significant cognitive benefit of aerobic training [45], and a most recent systematic review in 2017 showed no or limited effects [16], while large and specific benefits were found in 2003 [46] for the impact of physical training on executive function. Diverse and precise ways of evaluation and standardized assessment of cognition improvement in recent years may be one of the important reasons for the distinction.

### 2.3. Combine Training

The research on the combined effects of cognitive and physical exercises is rising; some research has reported that combining mental and physical exercises may be more effective than either alone and raised the point that cognitive decline is multicausal and onefold intervention will possibly remain insufficient [46, 47]. Exergaming is a typical type that combines physical exercise with interactive virtual reality, which provides cognitive stimulation when taking part in physical activities, for example, stationary cycling with virtual reality tours. Anderson-Hanley et al. confirmed that exergaming improved executive function and clinical status of mild cognitive impairment patients [15]. Meanwhile, Maillot et al. came to the similar conclusion that exergaming contributes to physical function and processing speed, which is the reflection of executive control capacity [48]. Simultaneous physical and cognitive exercises have greater potential for improving global cognition (working memory, episodic memory, and executive function) in a dose-response manner [49] and enhance frontal cognitive functions and gait [50] as well as neuroplasticity [15] while aerobic or resistance training has no advantage on episodic memory [51]. On the other hand, an interactive physical and cognitive exercise system (iPACES) uses neuro-exergaming wherein older adults engage in a physical exercise pedaling and steering a stationary bike along a virtue bike path to achieve their target heart rate and play a computer game theoretically designed to train cognition. Neuro-exergaming gained positive results in neuropsychological effects and executive function compared with exergaming or neurogaming groups, suggesting that combined exercises work well than focusing on either alone [52]. In addition, exergaming gains better engagements because of its enjoyment as it is often a challenge to motivate elders adhere to long-term exercises. However, it should be noted that when cognitive and physical exercises take part separately rather than simultaneously or interactively, no significant benefit of combined training [53, 54] would be found compared with either alone. Meanwhile, the amount of activity is important at the same time; a RCT in *JAMA* examined the synergetic effects of combined physical and mental activities in the elderly. For participants engaged in home-based mental activity plus class-based physical activity for 12 weeks and randomized to different groups according to the type of exercise intervention (mental activity: intensive computer or educational DVDs; physical activity: aerobic or stretching and toning), results demonstrated that there was no significant difference across all 4 randomized groups, suggesting that the amount and intensity of exercise is more important than the type of activity [55].

## 3. Other Influential Factors of Exercise Intervention

In addition to the type of exercise, a large amount of variation exists in the effect of exercise on cognition benefits. The duration of exercise intervention and the sex of participants are two major factors that need to be considered. Long-term and regular training has proven to be effective to improve performance on cognitive tasks such as executive function [56], long-term memory and attention [57], and has a positive impact on brain function via better cardiorespiratory fitness [40], increasing cerebral blood flow [58], and hippocampal volume [29]. However, even an acute, one-off exercise results in improvements on cognitive function. For example, participants who completed a 20-minute treadmill-based aerobic exercise for 5 days gained better cognitive control by flexible allocation of attention resources during the task [59]. Forty-eight subjects were allocated into a visuomotor accuracy-tracking task and drew the conclusion that acute exercise is related to long-term memory consolidation [60]. These findings are likely the results of the improvement of motor skill acquisition [61, 62] and motor memory [60], the development of cortical environment which would be more optimal for plasticity [63], and the reduction of inhibition in the motor cortex [64] after a single session of activity period of exercise. On the other hand, in the early 2003, Colcombe and Kramer have shown that the gender of the study participants could affect the outcome of exercise-induced cognitive benefits. Female became one of the predictors for positive results [46]. Studies with higher percentage of women participants showed greater beneficial effects on executive functions and no or marginally significant difference on episodic memory, visuospatial functions, word fluency, and processing speed [51]. Six months of large amount of aerobic exercise had sex-different effects on cognition; women gained better performance on various tasks of executive function than men [56]. Those results may relate to gender differences in brain-derived neurotrophic factor [51] and aerobic exercise-induced hypothalamic-pituitary-adrenal axis [51, 56]. In the meantime, many of the body diseases are also promoting factors of dementia especially the vascular dementia after cerebral infarction as those diseases compromised cognitive and brain health and consequently cause the risk of dementia. Therefore, preventing the happening of other chronic somatic diseases will also be one of the means to reduce the morbidity of dementia. Moreover, the relationship between exercises and diseases has got consistent results. High-intensity physical activities have been found to be faithfully associated with the reduction of all-cause mortality [65] and benefits in the secondary prevention of many chronic illness such as coronary heart disease, diabetes, and stroke. Meanwhile, the rehabilitation of diseases can also see the effect of exercise intervention [66]. Unfortunately, few individuals participate in aerobic or strengthening activities despite education from hospital and government. However, these studies identify exercise as a factor that reduces the risk of dying while its role in cognition has not been testified drastically.

## 4. Exercise Efficacy to Improve Cognition with Different Stages of Alzheimer's Disease

### 4.1. Mild Cognitive Impairment (MCI)

Mild cognitive impairment (MCI) represents a transition stage of cognitive functioning between normal aging and dementia, which represents newly acquired cognitive deficits that are more severe than individuals with the same age and educational background [67] or emerge subjective concern regarding change in cognition while functional abilities in everyday life are preserved [68, 69]. The cognitive dysfunctions including episodic and semantic memory [3, 14], working memory, and executive function [3] are discernible in MCI, and the initial symptom of MCI is a decline in memory (amnestic MCI) [67] such as familiar names [70]. Moreover, pathological changes such as amyloid plaques and agminated tau protein in regions of entorhinal cortex, parahippocampal gyrus, and subiculum in the medial temporal lobes can be tracked in MCI as well, although it may be not that severe compared with AD [71]. MCI patients have higher risk of developing AD, 40% of individuals diagnosed with MCI progressing to AD four years later [72]. However, there is a process for the transition from normal cognition to mild cognitive impairment and to Alzheimer's disease that with typical clinical symptoms. MCI patients could own normal cognitive functioning if with effective intervention while suffer rapid decline as the progression of the disease [73] and measures need to be taken at early stage as a result. Nevertheless, the thorniest problem is that there are yet no approved drug treatments to MCI. Cholinesterase inhibitors had a negligible effect in MCI [68] while *Ginkgo biloba* is ineffective in primary prevention [74]. Peterson et al. conducted a RCT that discusses vitamin E and donepezil in subjects with MCI. Vitamin E did not postpone the progression to Alzheimer's disease at any time node and proved to be invalid in patients with MCI, and donepezil significantly retarded the course of Alzheimer's disease during the first 12 months of the treatment year, but the rate of progression to AD was not reduced after 3 years [74]. However, a large body of literature suggests that exercise intervention may decrease the risk of Alzheimer's disease for MCI. Studies indicate that computerized cognitive training alleviates the cognition decline during the MCI [15, 75–77]. Law et al. designed a RCT aimed at evaluating the effects of a series of functional tasks on their role in MCI; results showed that general cognitive functions, memory, executive function, and the ability of daily life improved both in postintervention and 6-month follow-up [75]. Computer-based cognitive rehabilitation (CBCR) was assessed with MCI by Galante and colleagues, suggesting that CBCR plays a role in putting off the continuous progression of cognitive decline to AD [77]. Anderson-Hanley et al. confirmed that exergaming improved executive function and clinical status of mild cognitive impairment patient [15]. In addition, other studies discuss the efficiency of exercise training in MCI and AD which show that the improvement in AD is weak and limited compared with patients of MCI [76, 78]. A 4-week cognitive multicomponent rehabilitation resulted in significant improvements on activities of daily life and episodic memory in patients with MCI and nonsignificant increase with AD patients [78].

### 4.2. Moderate to Severe Alzheimer's Disease

During the past decades, quite a lot of studies work on Alzheimer's disease, but no major breakthroughs seem to be forthcoming. So far, there have been no curative therapies for this terminal disease including traditional and emerging drugs as it is almost impossible to reverse the severe cognitive impairment and neurodegeneration. Cholinesterase inhibitors (donepezil, rivastigmine, and galantamine) and memantine are the major pharmacotherapy. RCTs and reviews have demonstrated cognitive benefits of drug treatments while there seems minor clinical significance. A long-term donepezil RCT in Lancet showed small but significant cognitive improvement in MMSE. However, no benefits were seen in institutionalization rates or progression of disability after 3 years [79]. Memantine is the only drug approved by the US Food and Drug Administration in 2003. Placebo-controlled, double-blind, parallel group, randomized trials of memantine in people with different stages of Alzheimer's disease were analyzed and found that memantine has a small but significant benefit effect in moderate to severe AD but those with MCI did not gain that improvement in cognition and thus provide a reasonable choice for moderate to severe AD [80]. Therefore, the treatment goal of AD has turned to delay the progression of cognition decline and maintain the ability of daily life. Research on nondrug therapies has produced promising cost-effective strategies for dementia [81, 82], which improve behavior, mood, and the quality of life for families of patients with AD [82].

Meta-analysis aimed at comparing the effect of drug treatment and exercise intervention in AD, indicating that pharmacological therapy has inflexible impact on cognitive function while exercise has the potential to modify cognition dysfunction in AD as well [2]. Some experiments have established that exercise intervention of dementia is invalid especially in cognitive function [86] and behavioral disturbance [83]. However, cognition will decline rapidly if one is without exercise. A RCT in *JAMA* suggested that the physical function deteriorated during the year after exercise intervention regardless of group-based exercise or tailored home-based exercise while those receiving usual medicine care merely have a significantly faster decline [81]. Nascimento et al. obtained similar results that the experimental group showed a tendency for less reduction in neuropsychic disturbance and performance of instrumental activities compared to the control group who lacks exercise [84].

Meanwhile, intensive and long-term exercises were proven useful to physical functioning of home-dwelling [81] and institutionalized patients with AD [83], leading to relative better activities of daily life [83]. In addition, as dementia progresses, the weight loss, sarcopenia [81], and the decline in frontal cognitive functions [85] become more severe, connecting with a significant risk of falls, which is one of the important elements that result in disability, permanent institutional care, and mortality. Fortunately, multimodal exercise intervention has been identified effective in the improvement of frontal cognitive functions [50, 86] and proved to be an irreplaceable part of the rehabilitation to AD patients despite their severe cognitive impairment [87]. On the other hand, some studies take exercise as part of comprehensive intervention while results were controversial. Teri et al. suggested that exercise training plus behavioral management help improve physical function for patients with Alzheimer disease [88]. However, two years of home-based occupational therapy plus collaborative care did not obtain definite consequence on improving the rate of functional decline in patients with AD [42].

### 4.3. Normal Aging People

Along with the aging of the population and the expectation of the elderly to be physically and psychologically healthy, there is an imperative need to take effective interventions to improve or at least maintain cognitive function in normal aging people. Here, the normal cognitive decline associated with age which is different from MCI (beyond normally excepted cognitive decline) and dementia (cognitive decline in one of several cognitive domains) is discussed. A 6-week double-blind randomized placebo-controlled trail did not obtain positive results in semantic encoding by donepezil with cognitively normal elderly [89]. Other RCT on memantine showed no benefits on memory with age-associated memory impairment similarly [90]. Meanwhile, pharmacological interventions such as hormonal therapies, *Ginkgo*, and vitamins have not gain enough evidence for cognition prevention [91–94] as well. However, a large number of experiments have established the impact of nonpharmacological interventions on cognition. Cognitive training [23, 34, 95, 96] may acquire consistent memory benefit while physical exercise improves cognitive performance [37, 57, 97]. High-quality research suggested that cognitive training in cognitively normal elderly can be effective and durable. In this study, the neuropsychological gain was encouraging; the training effects were equivalent to a protection that the elders will not suffer from cognitive decline for 7 to 14 years [98]. Evidence above suggests that exercise intervention plays an important and irreplaceable role on the prevention of cognitive deterioration with healthy older people.

## 5. The Underlying Mechanisms of Exercise Intervention on Cognitive Improvement

Emerging evidence has indicated that exercise intervention does benefit to cognitive function, but the underlying molecular mechanisms remain unknown. Currently, AD is diagnosed through insoluble amyloid *β*-peptide (A*β*) in extracellular plaques and agminated tau protein in the intracellular neurofibrillary tangles by the detection of postmortem [3, 4]. As a result, it is crucial to clarify how exercise affects AD pathology. Studies of pathological change in transgenic AD mouse and structural imaging in human have provided some clues. Adlard and colleagues found that voluntary exercise reduced A*β* load in frontal cortex and hippocampus in transgenic AD mouse and the mechanism proved to be independent of classical A*β* degradation pathways for there was no change in the expression of the key enzyme neprilysin and insulin-degrading enzyme (IDE) [10]. However, short-term exercise activity decreased the proteolytic fragments of amyloid precursor protein (APP), supporting that exercise reduced A*β* by regulating the APP metabolism and the amyloid cascade, leading to the final result of learning and memory improvement [10]. On the other hand, the examination of the effects of environmental enrichment (large cages containing running wheel, colorful tunnels, and assorted toys) in transgenic AD mouse models showed controversial consequences in terms of amyloid deposition, with reduced A*β* levels and amyloid deposition in some investigations [11, 99] but neither decrease of amyloid or tau by studies of others [100, 101]. Therefore, exercise-induced cognitive improvement with no change on the level of A*β* may be associated with the mechanism independent of A*β* deposition. Moreover, other studies use radiological technology and serum biochemical markers to clarify the interaction between exercise and AD pathology in humans. Liang's group chose Pittsburgh compound B (PiB) detected through positron emission tomography (PET), and A*β* and hyperphosphorylated tau proteins (ptau) measured in cerebrospinal fluid (CSF) as biomarkers, and found that exercise was associated with reduced amyloid deposition and no significant changes in ptau [102]. This may be due to the participant of the research whom was in the early stage of cognitive decline while ptau is largely formed in the later course of AD [103]. Similar to animal studies, there are divergent results in the relationship between exercise and human AD biomarkers as well. Gidicsin's group confirmed that lifelong cognitive activity gives rise to cognitive improvement by a mechanism that is independent of A*β* deposition for there was no difference in the PiB retention [104].

In addition, it is important to note that exercise-induced modulation of ROS plays a role in better cognitive function and increased neurogenesis. As the central nervous system is too sensitive to the abnormal oxidative stress, studies confirmed that markers of lipid peroxidation are elevated in AD and MCI [9]. At the same time, studies have shown that the activities of antioxidant enzymes superoxide dismutase (SOD) and glutathione peroxidase (GPX) were increased in stem and corpus striatum after exercise [9]. It was also observed that the activity of neprilysin was increased, leading to the degradation of A*β* which may be responsible for the improvement of memory and cognition [11]. However, low levels of ROS may also impair cell function and break redox homeostasis while exercise results in the balance of oxidative challenge [105]. On the other hand, exercise is an important modulator of neurotrophins including BDNF, IGF-1, and vascular endothelial growth factor (VEGF). It has been suggested that BDNF is extremely critical in synaptic plasticity, neuroplasticity, and the development of learning and memory [106]. Not surprisingly, studies confirmed that the expression of BDNF could be upregulated by exercise and the downstream signaling pathway could also be regulated by exercise [13]. Other major neurotrophins elevated by exercise are IGF-1 and VEGF, which are critical for nerve growth and nutrition supply to the brain [13, 107]. Exercise-induced reduction of inflammatory markers (CRP, TNF-*α*, and IL-6) is probably one of its molecular mechanisms as well [108]. Furthermore, because cholinergic neuron damage is one of the causes of AD, the role of exercise in modulating cholinergic system also needs to be paid attention on. Results from some studies suggested that exercise may increase the level of acetylcholine [109] and the receptor density of dopamine and muscarinic [12], regulate neurotransmitter release in the hippocampus [110], and promote neuron proliferation [106].

## 6. Summary and Conclusion

Overall, exercise intervention is a promising, cost-effective treatment that benefits cognitive function and plays an important role on preventing the progression to dementia with mild cognitive impairment and older adults at risk of dementia. On the other hand, for patients with Alzheimer's disease, exercise intervention could slow down the rapid cognitive impairment and be part of comprehensive treatments. We summarized the exercise management of Alzheimer's disease (Table 1). However, to date, no standard strategy gets consistent agreement to different stages of cognitive decline. Though we have compared different types of exercise intervention, the duration of training sessions and the gender of the study participants in this review, no definite approach was gain still. Even if we have paid extensive attention to the mechanisms of exercise to cognitive improvement, including enhancing brain function, increasing cerebral blood, regulation of molecular biomarkers, balancing the oxidative challenge, reducing A*β* load and the levels of hyperphosphorylated tau proteins, and modulating cholinergic system, the detailed mechanisms of neural plasticity in dementia still require further tests to confirm. More experiments are needed to break through the treatment bottleneck of Alzheimer's disease.

## Figures and Tables

**Table 1:** Exercise management of Alzheimer's disease.

Type of exercise intervention	Stages of AD	Evidence
Cognitive exercise Cognitive training Cognitive stimulation Cognitive rehabilitation	Mild/moderate	Improve cognition Improve targeted cognitive abilities Improve self-reported instrumental abilities of daily living

Physical training Aerobic training Resistance training	Mild/moderate/severe	Improve physical function Improve executive function Improve spatial memory performance

Combine training Consecutive physical and cognitive exercise Simultaneous physical and cognitive exercise Interactive physical and cognitive exercise	Mild/moderate	Improve physical function Improve executive control capacity Improve global cognition Improve clinical status
